# Advanced Glycation End Products Effects on Adipocyte Niche Stiffness and Cell Signaling

**DOI:** 10.3390/ijms24032261

**Published:** 2023-01-23

**Authors:** Roza Izgilov, Alex Naftaly, Dafna Benayahu

**Affiliations:** Department of Cell and Developmental Biology, Sackler School of Medicine, Tel Aviv University, Tel Aviv 6997801, Israel

**Keywords:** AGEs, niche stiffness, adipocytes, carbonyl compounds, cell migration, adipogenesis, plasma-membrane stiffness, cytoskeleton reorganization

## Abstract

Adipose tissue metabolism under hyperglycemia results in Type II diabetes (T2D). To better understand how the adipocytes function, we used a cell culture that was exposed to glycation by adding intermediate carbonyl products, which caused chemical cross-linking and led to the formation of advanced glycation end products (AGEs). The AGEs increased the cells and their niche stiffness and altered the rheological viscoelastic properties of the cultured cells leading to altered cell signaling. The AGEs formed concomitant with changes in protein structure, quantified by spectroscopy using the 8-ANS and Nile red probes. The AGE effects on adipocyte differentiation were viewed by imaging and evidenced in a reduction in cellular motility and membrane dynamics. Importantly, the alteration led to reduced adipogenesis, that is also measured by qPCR for expression of adipogenic genes and cell signaling. The evidence of alteration in the plasma membrane (PM) dynamics (measured by CTxB binding and NP endocytosis), also led to the impairment of signal transduction and a decrease in AKT phosphorylation, which hindered downstream insulin signaling. The study, therefore, presents a new interpretation of how AGEs affect the cell niche, PM stiffness, and cell signaling leading to an impairment of insulin signaling.

## 1. Introduction

Advanced glycation end products (AGEs) are formed under conditions of hyperglycemia due to the reaction between proteins and high concentrations of glucose and its derivatives, the carbonyl compounds. The non-enzymatic glycation process results in covalent protein cross-linking, which increases the stiffness of the protein. We showed the alteration of structure and function of glycated modified serum albumin in [1]. The formation of AGEs and the consequent alterations in cellular activity and signaling suggest they play a role in a variety of metabolic disorders and, in particular, in diabetes complications. Notably, the AGEs may also be immunogenic and induce immune responses [2,3,4]. The adipocytes in fat tissue are responsible for lowering glucose levels. However, under conditions of hyperglycemia, the modification of cell function impairs both tissue and whole-body physiology, and results in problems with tissue remodeling and wound healing, as seen in diabetes [5,6,7]. An effect of glycation in a recent animal study conducted on mice fed with a high-fat diet (HFD) was evidenced in the formation of advanced glycation end products (AGEs), resulting in stiffening of the extracellular matrix (ECM), increased RAGE levels, and reduced insulin signaling [7].

In order to examine the changes caused by the formation of AGEs at the cellular level, we conducted a comprehensive study of adipocyte differentiation from their precursor cells. This included an analysis of the composition of the ECM proteins during adipocyte differentiation by MS/MS, as well as characterization of morphological changes of the cells, the function of the cell niche, and the cytoskeletal reorganization [8,9,10].

The importance of the adipocytes’ ECM and niche structure lies in the transduction of differentiation signals that affect the cells’ morphology and the modified shape converts undifferentiated fibroblast-like cells to round fat cells that accumulate lipid droplets (LDs). During this process, dynamic plasma membrane (PM) fluidity and cytoskeletal structure reorganization occur in response to biochemical and mechanical signals to the environment translated into cell signaling [9,10,11,12,13]. The ECM making up the cell niche is a complex of proteins, proteoglycans, and polysaccharides that define the cells’ niche signals and stiffness according to the protein content. Changes in the ECM provide a scaffold for adipocytes, where the stiffness and protein content affect and modulate processes, such as cell adhesion, migration, repair, and differentiation [14,15]. This is because the stiffness of the extracellular environment influences the signals and biomechanical forces transferred to the PM and cytoskeleton that activate intracellular signaling in adipocytes.

The current study was designed to demonstrate that AGEs, formed in a culture, caused the niche to become stiffer (measured by rheology) and affected the PM dynamic (measured by receptor binding using CTxB or endocytosis of nanoparticles (NPs)). The hyperglycation effect on cell migration was performed followed by time-lapse microscopy, and the cytoskeletal reorganization that occurred as the cells differentiation was measured. Mimicking the hyperglycation condition by the addition of intermediate carbonyl compounds led to reduced adipogenesis (measured by imaging and qPCR of adipogenic genes), attributed to an increase in ECM stiffness, which was generated by mechanical stress and physical stimulation. This affected membrane dynamics and also altered insulin signaling, as measured by a decrease in protein kinase B (AKT) phosphorylation, which mediates insulin signaling, and this results in insulin resistance (IR), as in type 2 diabetes (T2D) [16,17]. In this context, the consumption of high concentrations of sugars and carbonyl compounds might be a cause for the pathophysiology as recognized in diabetes.

## 2. Results

**Figure 1:**
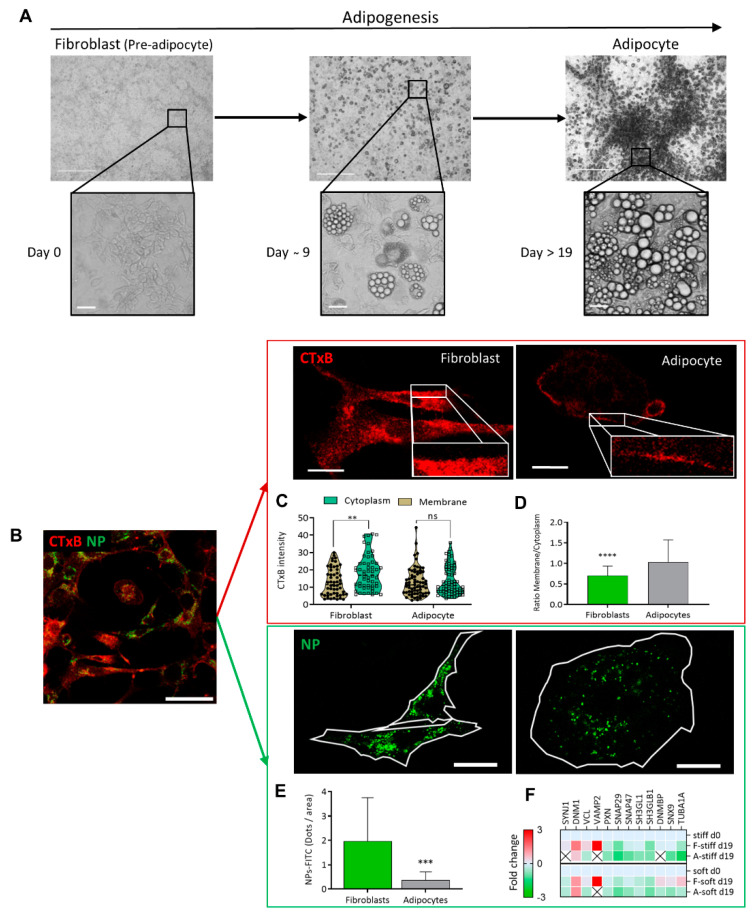
Endocytosis differences between fibroblast and adipocytes (**A**) Adipogenesis follow-up from fibroblast (pre-adipocyte) into adipocyte viewed by phase contrast micrograph at (×40 and ×400) magnification. Scale bar = 125 µm. (**B**) Immunofluorescence staining for the expression of CTxB binding (red) or NP-FITC (green) uptake was analyzed at single-cell resolution (B-E; scale bar = 20 μm). (**C**) CTxB intensity quantified in the cell membrane and cytoplasm of fibroblast and adipocytes. (**D**) The intensity of the membrane/cytoplasm ratio was compared between fibroblasts (n = 54) and adipocytes (n = 68). (**E**) NP–FITC internalization was measured at single cell fibroblasts (n = 16) or adipocytes (n = 18) per FOV. (**F**) Heat map from an MS/MS analysis for endocytosis-related proteins in fibroblasts and adipocytes in stiff/soft growing substrate conditions. (ns *p* > 0.05, ** *p* < 0.01, *** *p* < 0.001, **** *p* < 0.0001).

**Figure 2:**
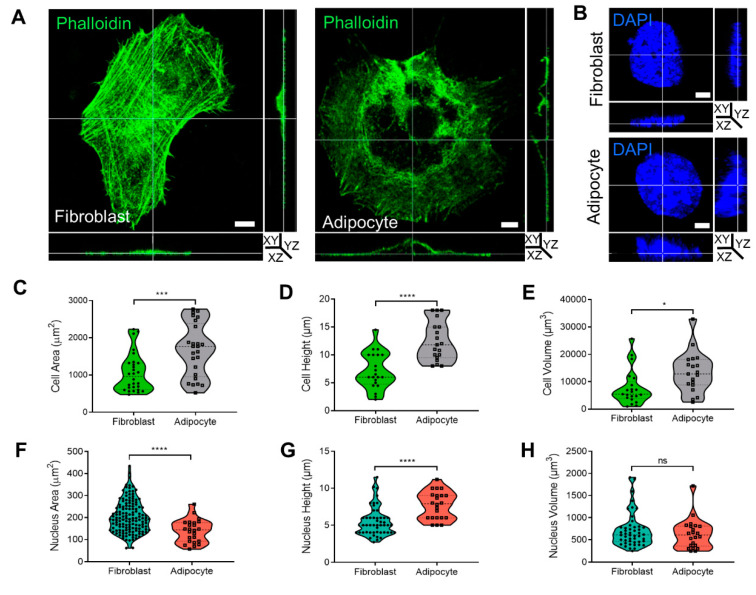
Cell shape, cytoskeleton, and nucleus measurement in 3D during adipogenesis. (**A**,**B**) Three dimensional confocal and Z stack micrograph of cytoskeleton’s actin staining (phalloidin-FITC) or nuclei (DAPI) (×630 magnification) on images of YZ and XZ orientation. (**C**,**F**) The measurement of cells (n = 28, fibroblast; and n = 25, adipocyte) and nuclei (n = 143, fibroblast; and n = 24, adipocyte) area. (**D**,**G**) Cells and nuclei height quantification was detected from the bottom to the top visible actin and DAPI staining (cells: n = 21, fibroblast, and n = 19, adipocytes); nuclei (n = 49 and n = 22, respectively). (**E**,**H**) Cells and nuclei volume quantification (n = 21, fibroblast; n = 19, adipocytes); nuclei (n = 48; n = 22, respectively). Data were presented as mean ± SD and statistics were analyzed by a two-tailed, unpaired Student’s t-test (ns *p* > 0.05, * *p* < 0.05, *** *p* < 0.001, **** *p* < 0.0001).

**Figure 3:**
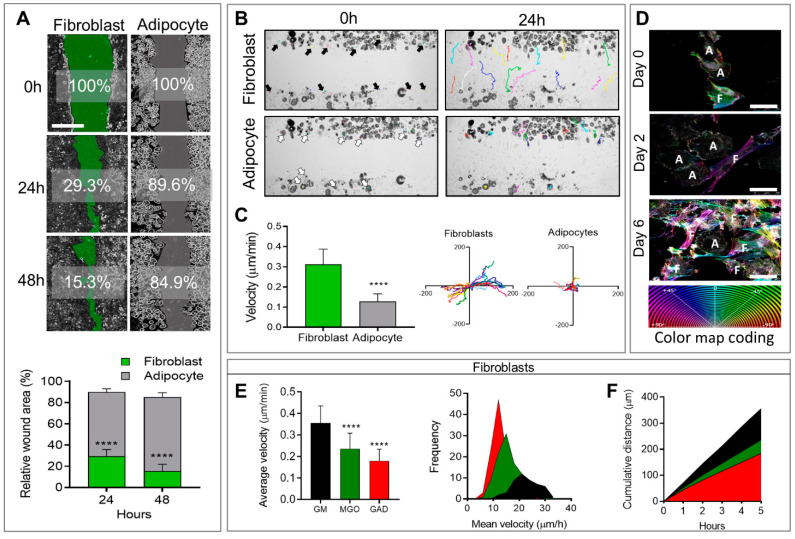
Wound healing and cell motility follow-up on live imaging. (**A**) Wound healing assay of fibroblast (green) and adipocytes (grey) follow-up over 48 h for % of wound area remaining to be closed (scale bar = 650 µm) (upper panel). The relative wound area for fibroblasts (n = 14) and adipocytes (n = 8) was quantified after 24 h and 48 h (lower panel). (**B**) Phase-contrast images; fibroblasts (black arrows, multicolor lines), and adipocytes (white arrows, multicolor dots) follow distance after 24 h. (**C**) Single-cell velocity of fibroblasts (n = 20) and adipocytes (n = 20) represented by a µm/min for over 24 h (left). Single-cell migration representation of a trajectory plot evaluating the accumulating distance, normalized to a common starting point (right). (**D**) The high-resolution images on days 0 to 6 illustrate the association between actin reorganization and cell motility (F–fibroblast, A–adipocyte) by a color map of HSB status (scale bar = 50 µm). The effect of hyperglycemic conditions on cell motility: (**E**) Average velocity quantification of individual cells was measured in untreated fibroblasts (n = 49, black), MGO (n = 98, green), and GAD (n = 106, red) treated cells (left). Frequency distribution of mean velocity range in treated groups (right). (**F**) Cumulative distance quantification displays the total cell’s distance over 5 h. Two-way ANOVA used for (**A**), Student’s unpaired *t*-test for (**C**), one-way ANOVA with Sidak’s multiple comparisons test (**B**), and Games–Howell multiple comparisons test (**D**); average velocity (mean ± SD). (**** *p* < 0.0001).

**Figure 4:**
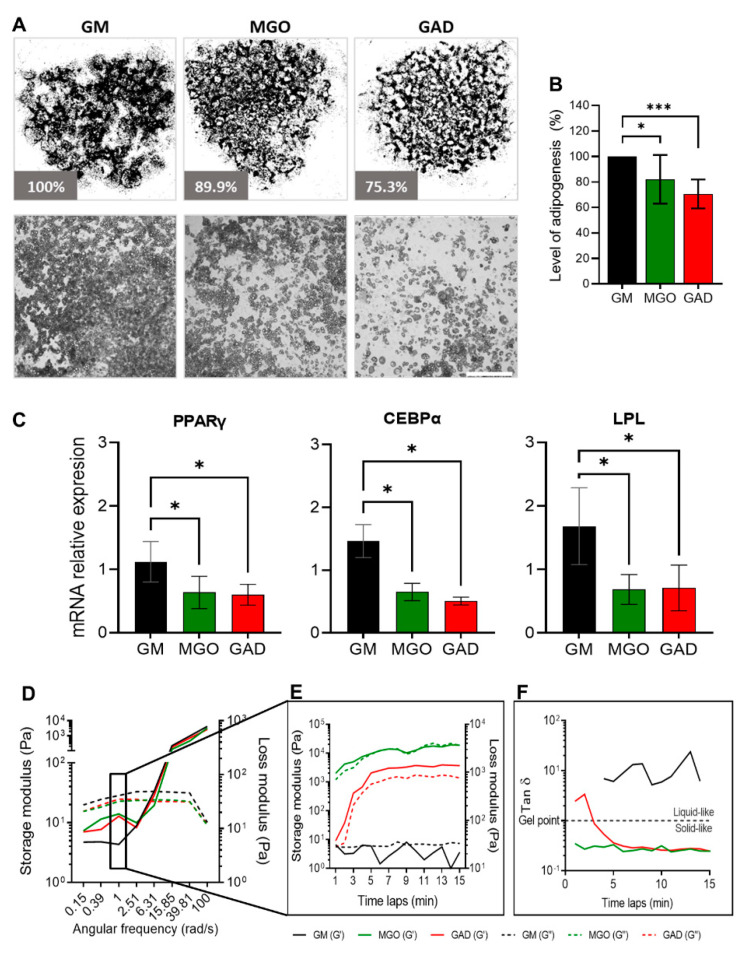
Adipogenesis under various hyperglycemic conditions, gene expressions, and stiffness changes. (**A**) LOA presented as a whole well stitching percentage analysis compared to GM of MGO- and GAD-treated cultures. Phase contrast representative culture images at ×40 (lower panel; scale bar = 650 µm). (**B**) LOA quantification analysis of MGO and GAD-treated adipocytes, compared to GM (n = 7). (**C**) The mRNA level of PPARγ, CEBPα, and LPL relative to actin in MGO and GAD-treated adipocytes, compared to control (GM, n = 4). One-way ANOVA ± SD. (**D**) Rheology measurements of GM-control (black), MGO (green), and GAD (red) measuring the G’ (solid lines) versus G” (dash lines) as a function of angular frequency sweep between 0.1–100 rad/s. (**E**) Time-dependent measurement of a G’ versus G” was conducted under a constant frequency over 15 min. (**F**) Gel point (Tan δ) measured by the loss and storage modulus ratio over time. (* *p* < 0.05, *** *p* < 0.001).

**Figure 5:**
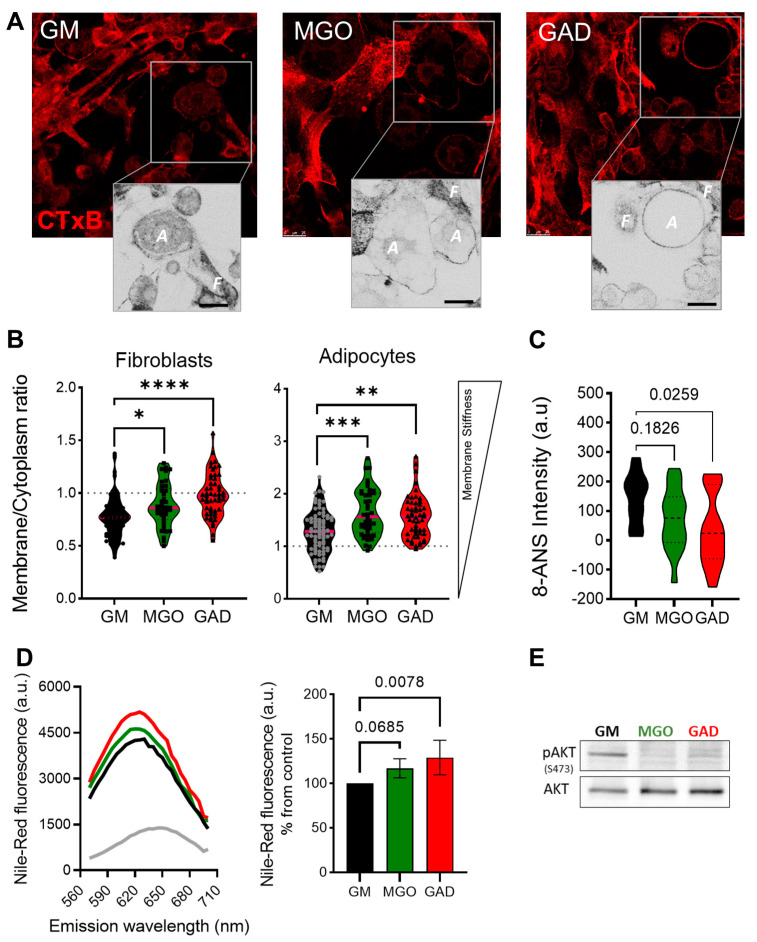
Membrane stiffness and signaling alteration in the MGO and GAD-treated cultures. (**A**) Confocal images of CTxB labeled cells (F–fibroblast, A–adipocyte), enlarged image highlighted with ICA LUTs to emphasize the cells’ membrane intensity (scale bar = 25 µm). (**B**) Quantification of membrane/cytoplasm CTxB expression ratio at the single-cell level, analyzed in fibroblasts and adipocytes (n = 40–80 cells per group). (**C**) The 8-ANS spectroscopy of the GM culture lysate compared to MGO and GAD-treated cells (n = 6). (**D**) Spectroscopy of treated adipocytes in the presence of Nile red: fluorescence spectrum with blue shift peak (right), and peak value quantification (left, n = 4). (**E**) Western blot analysis of phosphorylated AKT protein (pAKT^ser473^) compared to total-AKT in GM, MGO and GAD-treated adipocytes. Results presented as mean ± SD, statistically analyzed by one-way ANOVA. (* *p* < 0.05, ** *p* < 0.01, *** *p* < 0.001, **** *p* < 0.0001).

Adipogenesis is a process by which fibroblast-shaped pre-adipocytes (hereafter fibroblasts-F) differentiate into adipocytes (A), and is accompanied by an accumulation of lipid droplets (LDs) [9,18]. In this study, we used live imaging to follow up processes during adipogenesis, PM receptor binding and NP-FITC internalization (Figure 1), changes in cytoskeleton reorganization (Figure 2), wound closure, cell motility (Figure 3), alterations in cell/niche stiffness (Figure 4) and cell signaling (Figure 5).

The morphology of cell differentiation and lipid droplet accumulation during the culturing is seen in Figure 1A on phase contrast microscopy. The cultures were investigated with high-resolution confocal microscopy, to compare the cell membrane dynamics of fibroblasts and adipocytes at the same culture (Figure 1B). Two model systems were used, where the first involved binding of a CTxB fluorescence probe (red) to ganglioside M1 (GM1) and following receptor-mediated endocytosis (red) (Figure 1C,D), and NP–FITC internalization (green, Figure 1E,F). The binding of the CTxB (red) to ganglioside M1 (GM1) was measured, and the ratio of fluorescence labeling in the membrane to the cytoplasm (M/C) was calculated as a measure of membrane dynamics/stiffness. The results indicated a 1.4-fold higher CTxB intensity in the cytoplasm of fibroblasts than in the membranes (5.39 ± 2.08; *p* < 0.003) (Figure 1C). In contrast, the CTxB membrane/cytoplasm fluorescence ratio in adipocytes was around 1 compared to 0.6 in fibroblasts (Figure 1D), indicating less permeability of CTxB in the adipocytes. The second assay of non-receptor-mediated endocytosis, using nanoparticles (NP–FITC) allowed quantification of the NPs’ internalization, results with a 5.3-fold higher intensity of NP-FITC in fibroblasts than in adipocytes (*p* = 0.0007, Figure 1E). Complementary to the endocytosis process, we compared protein expression, based on MS/MS analysis. The analysis indicated the protein expressed was related to the cells’ differentiation levels and the stiffness of their substrates [9]. The comparative MS/MS analysis of fibroblasts and adipocytes, presented as a heat map of the proteomic data, confirmed changes in the expression of proteins related to endocytosis, exocytosis, PM fusion, and docking. These proteins included Synaptojanin-1 (Synj1), Vesicle-associated membrane protein-2 (VAMP2), and Dynamin Binding Protein (DNMBP). Some proteins were below the level of detection in adipocytes; suggesting that the machinery related to endocytosis has a lower functionality in adipocytes than in fibroblasts (Figure 1F). Thus, the MS/MS results related to endocytosis and regulation of membrane dynamics supported the presented imaging revealed by CTxB or NP–FITC endocytosis.

The cytoskeleton organization plays a role in membrane dynamics and endocytosis. In this context, we previously reported 2D confocal images demonstrating that morphological changes to adipocytes were related to cytoskeleton rearrangement into a rim around the plasma membrane [9,10]. To further investigate the cytoskeletal rearrangement during adipogenesis, cells were stained with phalloidin, and the cytoskeleton and cell nuclei were visualized by confocal microscopy. Three-dimensional analysis of fibroblast and adipocyte cells (Figure 2A,B, Supplementary Files S3 and S4) revealed that adipocytes were 63% larger (1672.1 ± 142.8 µm^2^) than fibroblasts (1059.8 ± 97.5 µm^2^, *p* < 0.001; Figure 2C). Similarly, the diameter (cell height) of the adipocytes was 57% higher (12.4 ± 0.8 µm) than that of fibroblasts (7.1 ± 0.7 µm, *p* < 0.0001; Figure 2D), and the volume was 58% larger (13,360.9 ± 1691.7 µm^3^) than that of fibroblasts (7748.3 ± 1406.7 µm^3^, *p* < 0.05; Figure 2E). There was a significantly smaller area of nuclei detected in a FOV of adipocytes (139 ± 10.5 µm^2^) than in fibroblasts (204.5 ± 6 µm^2^ *p* < 0.0001; Figure 2F), with average height values of 7.6 ± 0.5 µm^2^ in adipocytes and 5.1 ± 0.5 µm^2^, *p* < 0.01 in fibroblasts (Figure 2G). Overall, the measured nuclear volume of the fibroblasts was 712.3 ± 380.2 µm^3^ and for adipocytes measured 629.7 ± 335.4 µm^3^ (Figure 2H).

In addition, to affecting morphological changes and cytoskeletal reorganization, cell differentiation also affects cellular motility and migration capacity, measured by means of a wound healing assay followed by live imaging follow-up. Live imaging over 48 h after the generation of a wound (scratch) revealed that adipocytes generally remained motionless and just moved around “themselves”, while fibroblast migration was fast and spread quickly in different directions (Figure 3A, Supplementary Files S1 and S2). This translated to a significantly faster rate of wound closure in fibroblasts (29.3 ± 1.7%) after 24 h with an additional 15.3 ± 1.8% after 48 h, than in adipocytes (89.6 ± 1.1% and 84.9 ± 1.5%) over 24 and 48 h, respectively, with *p* < 0.0001 for both time points (Figure 3A). The cellular velocity was quantified at a single cell level over 24 h at intervals of 20 min. The colored lines in the photographs taken after 24 h (Figure 3B) represented the same individual cells shown by arrows in the images taken at time zero. These velocity results confirmed that adipocytes generally remain in the same place (0.128 ± 0.0087 µm/min), while fibroblasts move three times faster (0.319 ± 0.017 µm/min, *p* < 0.0001; Figure 3C). The distance was measured on the XY orientation, and each color represented an individual cell (Figure 3C). These results demonstrated that fibroblasts, but not adipocytes, were capable of closing a wound in the same culture.

A novel aspect of this study was the effect of AGE formation in the presence of an excess of carbonyl compounds (MGO and GAD). AGEs that accumulated affected the cells and, specifically, the stiffness of their niches. A culture treated with carbonyl compounds induced AGE formation and was studied for its effect on cells’ fates and functions. Adding carbonyl compounds to cultures affected cells’ motility. Since the adipocytes did not move much (Figure 3), we focused on the effect of hyperglycation on fibroblast motility and migration when exposed to MGO, and GAD for 10 days in a culture. Live imaging allowed following the cell dynamics, revealing that the treatment inhibited fibroblast motility, measured at a single-cell level to measure the cells’ velocity and distance. An average velocity of control (GM) was 0.36 ± 0.08 µm/min that became lower under glycated conditions. Under MGO treatment, we monitored a velocity of 0.24 ± 0.07 µm/min, (*p* < 0.0001). Cells treated with GAD were slower with an average velocity of 0.18 ± 0.05 µm/min (*p* < 0.0001, Figure 3E), and both were slower than cells’ motility grown under GM. Quantification of the frequency distribution versus the mean velocity (µm/h) revealed a significant shift in MGO- and GAD-treated fibroblasts, compared to GM. GAD treatment resulted in the highest peak with a frequency of 12 µm/h, compared to 21 µm/h in the GM group (Figure 3E). Five-hour cumulative distance measurements revealed significantly more movement for fibroblasts grown with GM (355.28 ± 11.41 µm), MGO (236.25 ± 7.28 µm; *p* < 0.0001), or GAD (184.29 ± 5.73 µm; *p* < 0.0001) (Figure 3F). These results indicated that adding carbonyl compounds to cultures reduced fibroblast motility.

Adipogenesis was assessed by treating cultures with the carbonyl compounds viewed at the macro-level of cultures and the LOA under the different treatments (Figure 4A). Both carbonyl compounds significantly decreased the adipogenesis level by about 30% (MGO: 82.1 ± 19.1%, GAD: 70.6 ± 11.4%) (Figure 4B). A reduced LOA was complementary to the alteration in the expression of adipogenic marker genes; PPARγ, LPL, and CEBPα levels decreased in MGO- and GAD-treated cultures (Figure 4C).

Furthermore, we noted that carbonyl compounds altered the stiffness of the cells and ECM i.e., creating stiffer cells’ niche during adipogenesis, measured with a rheometer (Figure 4D–F). Considering the effect of AGE formation leading to an increase in stiffness of cultured adipocytes under hyperglycemic conditions represents a novel approach. We, therefore, measured the storage and loss modulus of cells and ECM lysates of differentiating adipocytes grown in the presence of MGO and GAD and the results showed a crossover between the storage (solid lines) and loss (dash lines) modulus at a frequency of 6.3 rad/s, which suggested a similar relaxation time in all the cell samples (Figure 4D). A comparison of the storage modulus at a frequency of 1 rad/s measured over 15 min was performed to examine the differences between treatments. The results revealed that lysates treated with GAD and MGO were 100-fold stiffer than GM-treated cultures (Figure 4C–E), emphasizing the viscoelastic properties displayed. MGO-treated cells displayed a smaller lag between the storage and loss modulus than GAD-treated cells, which might indicate a less gelatinous state (Tan δ). Although no treatment-related differences in Tan δ were detected, MGO- and GAD-treated cells had a higher value of G’ compared to G”, while adipocytes grown in GM appeared more liquid-like (Figure 4E). We could, therefore, conclude, with the novel concept, that exposure to MGO or GAD elevated the cellular stiffness and, consequently, the biomechanics of the cells. These results further prompted the view that the effect of cells’ membrane stiffness would also be expected to have a substantial impact on signal transduction.

Next, we labeled the live cells to measure the CTxB binding (as shown in Figure 1). This was done through the addition of MGO and GAD, which were then analyzed using confocal microscopy. The images acquired were converted into ICA LUTs to highlight the intensity, and enlarged pictures are displayed underneath the original images in Figure 5A. The CTxB intensity expression quantified the ratio between the values in the membrane/cytoplasm (M/C). The results indicated a higher ratio for fibroblasts grown with MGO and GAD than for GM-treated controls. Figure 5B demonstrates that the CTxB intensity ratio was predominantly in the fibroblast cytoplasm (<1), rather than in adipocytes (>1). The M/C ratio for adipocytes grown with GM was lower than for cells treated with MGO, and GAD (Figure 5B). The high M/C ratio (>1) indicated more CTxB in the PM than in the cytoplasm, i.e., a reduction in the internalization of CTxB in adipocytes treated with the carbonyl compounds. In parallel, spectroscopic analysis in the presence of an 8-ANS probe in adipocytes’ cell lysates indicated a 1.5 to 2-fold change in the pattern of cells exposed to hyperglycemic conditions i.e., a higher level of AGEs creation (Figure 5C). Results on glycated conditions obtained by spectroscopy, in the presence of Nile-Red, showed blue shift spectra and fluorescence elevation in the treated cultures (Figure 5D). Both spectroscopy assays, which indicated the alteration under glycation, led us to analyze insulin signaling by measuring AKT phosphorylation. MGO and GAD treatment decreased phosphorylated AKT (pAKT^ser473^) in adipocytes and was indicative of insulin tolerance, which validated the notion of changes in cellular insulin sensitivity (Figure 5E).

## 3. Discussion

This study brings a new view that introduces the relationship between adipocyte differentiation, signaling, cell activity, and alterations in the stiffness of cells, and the ECM landscape i.e., the cell niche. We assumed that alterations in the stiffness of the cells and ECM affect the mechanical forces experienced by the cells and, thereby, modulate cellular signaling and functioning [7,13]. The changes in stiffness were due to crosslinking between sugars and amino acids of proteins, i.e., the generation and accumulation of AGEs. The results presented here describe how the AGEs formed in culture in response to hyperglycemia affecting cellular behavior and the ECM landscape. The 3T3-L1 cell model was used by us, and has been used by others, to demonstrate the alterations in ECM protein content that occur during the differentiation of mesenchymal cells [9,14,18,19,20].

During adipogenesis, cells change shape from undifferentiated elongated fibroblasts to round adipocytes. These morphological changes are accompanied by cytoskeletal reorganization where the linearly organized actin filaments in the fibroblast cytoskeleton are modified and shortened to become the spherical fibers arranged as a rim around the plasma membrane seen in adipocytes [9,18,21,22]. The changes in actin, a cytoskeleton protein organization related to the altered proteins in the ECM, such as collagen types 4, 6, and 18, as well as lamin, provide the signals to differentiating adipocytes [9,15,23,24,25,26,27]. Further evidence for the important role of the cytoskeletal was provided by a study that demonstrated an association between F-actin and Myh10 in the actin–myosin complex. Myh10 accelerates interactions with F-actin [9,10,28], thereby contributing to F-actin assembly, and generating the strength to resist extension and contractile by binding to actin filaments [29]. Notably, the knockdown of Myh10 affects cells’ migration and reduces adipogenic activity [9,10]. In addition to the changes in cytoskeleton and ECM content, we also investigated cellular motility. As expected, the results confirmed that fibroblasts are active and motile with a capacity for migration, while adipocytes tend to remain static with minimal movement around themselves (Figure 3). Fibroblast proliferation and migration has an important role in the active process of wound healing [30,31]. Interestingly, the wound healing capacity of fibroblasts was impaired under hyperglycemic conditions, with significant reductions in velocity, motility, and cell migration, compared to untreated fibroblasts (Figure 3). Such effects were shown for fibroblasts isolated from diabetic cells compared to control [6,32], a phenomenon that could explain the wound healing difficulties seen in patients with diabetes [5].

The relation between the cytoskeleton and membrane dynamics/stiffness was studied by a receptor-mediated process (CTxB binding in live cells) and non-receptor-mediated, NP–FITC endocytosis. The lower level of CTxB internalization to the cytoplasm in adipocytes and the ratio in the membrane/cytoplasm indicated that the plasma membrane was less dynamic than in fibroblasts (Figure 1). The underlying cellular activity of endocytosis is regulated by the cytoskeleton related to actin organization and, therefore, to the cell membrane flexibility. In this context, we previously used atomic force microscopy (AFM) and interferometry microscopy to demonstrate that adipocytes become stiffer with the accumulation of LDs [33]. The movement and migration necessary for wound healing and cell motility in fibroblasts depend on organized actin filaments that are not present in adipocytes (Figure 2). In addition, hyperglycemic conditions also reduce the motility in fibroblasts (Figure 3), probably by a similar effect of glycation as was recently demonstrated by us with serum albumin [1]. Further, the effect of glycation on adipogenesis level and the cells’ niches was studied here for the first time, mimicking a response to hyperglycemia (in the presence of carbonyl compounds) (Figure 4). Adipogenesis inhibition after exposure to treatment with MGO and GAD (Figure 4A) was also confirmed by the decrease in the expression of the key genes in adipogenesis, such as PPARγ, LPL, and CEBPα (Figure 4B) [34].

Other changes involving alteration in the cell/ECM viscoelasticity, detected by rheology (Figure 4C–E), showed an increase in stiffness in culturing and generation of the solid–like “material” presence of MGO and GAD i.e., formation of AGEs is a fast process that occurs within days and the cells are in a stiffer ECM. Notably, spectroscopy assays, using 8-ANS, confirmed the formation of AGEs after treatment with these compounds (Figure 5D), shown also in the spectral shift in the presence of Nile red (Figure 5E). These results were also supported by other studies [35,36].

The experiment showed an alternate membrane stiffness during adipogenesis for cells treated under glycation, both fibroblasts and adipocytes, studied by the CTxB binding, which confirmed that cells treated with carbonyl compounds were stiffer than untreated cells (Figure 5). This was also shown by the rheology analysis (Figure 4). In addition, we recently showed, by means of the rheology method, that adipose tissue from mice fed a high-fed diet also increased in stiffness [7]. The result of a reduction in adiposity was in accordance with previous studies demonstrating that hyperglycemia decreased differentiation in different cell types and increased endothelial cell membrane stiffness and could, at least partially, be attributed to cytoskeletal actin reorganization [37,38,39,40]. Notably, regarding the effect of such glycation conditions, studies support impact on decreased differentiation, and, herein, we also show another effect of hyperglycemia is a decrease in AKT sensitivity, as evidenced by decreased levels of protein phosphorylation (Figure 5E).

Taken together, the in-vitro model of adipogenesis treated under hyperglycemic conditions indicated the ability of carbonyl compounds to form AGEs that increase cell niches and PM stiffness. This process changes the cellular interaction with the ECM, which, consequently, affects cytoskeletal reorganization. The biomechanical implications of a stiffer cell niche translate to changes in signals transferred to the resident cells and consequent changes regarding cell fate and function, resulting in a vicious cycle that reduces adipogenesis. The adipose cell serves as a metabolic sensor and studies at the tissue level further advance our understanding of basic physiological mechanisms that can explain metabolic and clinical physiology.

**Figure 6:**
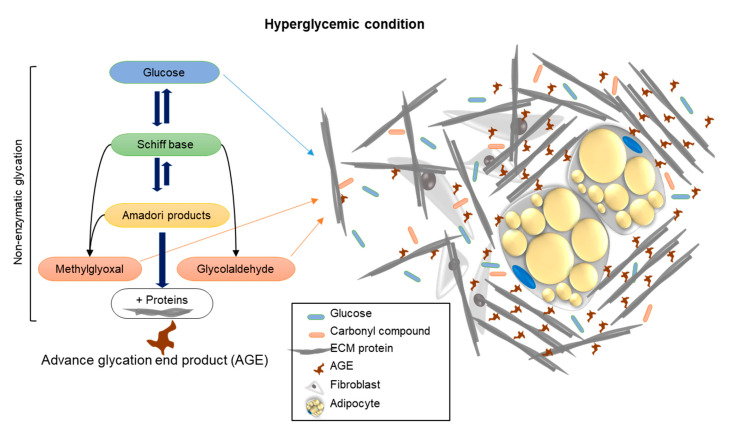
A schematic illustration showing the mechanism of how carbonyl compound-induced hyperglycemia affects the cell’s niche stiffness through the formation of AGEs leading to changes in protein structure and functionality.

Summary: This study brings a new aspect to the investigation of unique approaches and new viewpoints that study the effect of AGE formation, by examining the stiffness of the cell niche by means of rheology. Hyperglycemia, through treatment with carbonyl compounds such as MGO and GAD, accelerates the production of AGEs through protein crosslinking, as demonstrated in the schematic illustration (Figure 6). The conformational changes of proteins affect/damage their structures and functions. An increase in the amount and density of ECM components and the consequent rise in niche stiffness generates biomechanical forces, which, in turn, provide alternative cell signaling and reduce cell differentiation. We can conclude that an elevation of glycated conditions in the cell niche increases the formation of AGEs, which influence the cells’ cytoskeletal reorganization, PM functionality, and cell signaling.

## 4. Materials and Methods

### 4.1. Cell Culture and Level of Adipogenesis (LOA)

The 3T3-L1 pre-adipocytes (fibroblasts) cells (American Type Culture Collection, Manassas, VA, USA) were cultured for differentiation, as previously described in [9]. Cells were cultured on a glass coverslip 20 × 20 mm and placed in a 6-well plate (Jet Bio-Filtration Co., Guangzhou, China) for immunofluorescence. Cells were plated in a 6-well plate for the wound healing assay or biochemistry analysis. Cells were cultured with growth medium (GM) containing Dulbecco’s modified Eagle’s medium (4500 mg/dL D-glucose), 10% fetal bovine serum 1% L-glutamine, 0.1% penicillin-streptomycin and 0.5% 4-(2-hydroxyethyl)-1-piperazine ethane sulfonic acid (HEPES). Differentiation was induced when the cultures reached sufficient confluency (~90%) by applying a differentiation medium (DM); consisting of GM supplemented with 100 IU/mL insulin (Biological Industries, Cromwell, CT, USA), 1 μM dexamethasone (Sigma-Aldrich, St. Louis, MO, USA), and 400 μM 3-isobutyl-1-methylxanthine (IBMX; Sigma-Aldrich) for two days. After that, DM was replaced with a supporting medium (SM) consisting of GM supplemented with 100 IU/mL insulin, and the SM was changed twice a week. Differentiating cultures were supplemented with methylglyoxal (MGO, M0252, 0.001 mg/mL) or glycolaldehyde (GAD, G6805, 0.01 mg/mL) (Sigma-Aldrich) for an additional 10 days. Cultures were incubated at 37 °C in a humidified atmosphere, containing the 5% CO_2_. Images of cultured 3T3-L1 cells throughout differentiation were taken to follow the cells’ growth, morphology, and LD accumulation using a phase contrast microscope. We followed adipocyte differentiation and conducted follow-up on live unstained cultures, LOA was visualized with the ×40 magnification, allowing mapping of the cultures, and images were taken by optical phase-contrast photographs (EVOS FL Auto-2 Microscope, Invitrogen, Waltham, MA, USA). Stitching of images was used to calculate the LOA, as previously described [41].

### 4.2. Wound Healing and Cell Motility Assays

(i) The wound healing assay was analyzed in the culture following a scratch formed with a pipet tip, immediately photographed at the macro-level under ×40 magnification. Live imaging follow-ups photographed in 20 min intervals were observed on a phase contrast microscope (EVOS FL Auto 2, Invitrogen) and followed the relative closure of the gap. (ii) Motility analysis on cultures photographed was analyzed under ×40 and ×200 magnification, allowing measuring of the distance and velocity at the single-cell level analyzed by ImageJ software.

### 4.3. Cell Staining and Microscopy Imaging

(i) Cells were incubated with GM1 ganglioside binding Cholera Toxin Subunit B, Alexa Fluor™ 647 Conjugated (CTxB-647; Thermo Fisher Scientific, Waltham, MA, USA, C34778) for 5 min and immediately fixed with 4% formaldehyde containing 0.03M sucrose in phosphate-buffering for 10 min at room temperature (RT), and then washed in 1% TBS-T with 0.5% Triton. (ii) F-actin stained with phalloidin–fluorescein isothiocyanate, labeled (Phalloidin-FITC; Sigma-Aldrich, P5282) and mounted with Fluoroshield™ mounting medium containing 4′, 6-diamidino-2 phenylindole (DAPI; 17985-50; Electron Microscopy Sciences, Hatfield, PA, USA). The three-dimensional (3D) analysis using Z-stack images at 0.5 μm intervals from the bottom to the top of the cells allowed measurement and quantification of the actin reorganization changes, filament length and orientation, and the volume changes of cells and their nuclei during adipogenesis [9]. Cell and nucleus volume was measured by Surpass Scene on Imaris software (Oxford Instruments, Abingdon, UK) (iii) Silica nanoparticles 120 nm-FITC Conjugate (NP-FITC) [42] were used to measure endocytosis when incubated in cultures overnight at 37 °C, and then the cells were fixed. Images were taken with confocal microscopy (Leica SP5; Leica, Wetzlar, Germany) at ×630 magnification.

### 4.4. Protein Extraction and Analysis

Cells from cultures were lysed and applied for western blot as described in [10]. The primary antibodies used were actin (mouse, MP-691001/2), pAKT^ser473^, and total AKT (#9271, #9272, Cell signaling technology, Danvers, MA, USA). The secondary antibody that was used was donkey anti-rabbit (711-035-152; Jackson ImmunoResearch Laboratories, West Grove, PA, USA). The signal was detected with chemiluminescent substrate (SuperSignal^TM^ West Pico PLUS kit, 34578, Thermo Scientific Scientific, USA), exposed and quantified digitally on Fusion FX7 (Vilber Lourmat, Collégien, France).

### 4.5. Spectroscopy Measurements

Cultured cells were collected, washed with ice-cold PBS, and lysed in 25 mM Tris pH 7.5, 150 mM NaCl buffer, 0.1% SDS, 0.5% sodium deoxycholate, and protease inhibitors (phenylmethylsulfonyl fluoride, PMSF 1 mM; 1-chloro-3-tosylamido-4-phenyl-2-butanone, TPCK, 10 μg/mL). Fluorescent spectroscopy was conducted as follows: (i) 1-anilino-8-naphthalene sulfonate (8-ANS, A1028; Sigma-Aldrich) was added to cell lysate at a final concentration of 120 µM in borate buffer (200 mM, pH = 8.7), and incubated for 5 min in 96-well black microplate. Fluorescent absorbance was measured at Ex:370 nm/Em:470 nm. (ii) Nile Red (NR, 72485, Sigma-Aldrich) dye was added to cell lysate at a final concentration of 6 µM in PBS in a 96-well black microplate. Measured emission spectra performed at 570–700 nm. Fluorescence quantification was performed at Ex:540 nm/Em:620 nm. The plate was read using a microplate Spectra MAX M5 (Molecular Devices, San Jose, CA, USA) [1].

### 4.6. RNA Isolation and qPCR

As previously described [10], total RNA was extracted from 3T3-L1 cells using Trizol reagent (Bio Tri RNA; Bio-Lab Ltd., Jerusalem, Israel) and reverse transcribed to cDNA using a high-capacity cDNA reverse transcription kit (Applied Biosystem, Waltham, MA, USA). Transcript levels were measured with SYBR green (Applied Biosystem, Waltham, MA, USA) using STEPONE plus system (Thermo Fisher Scientific, Waltham, MA, USA). All data were normalized to actin by the delta-delta Ct method [43]. For qPCR amplification, we used the primers for the following genes:

(1) PPARγ–(F) ATTCTCAGTGGAGACCGCCC; (R) GGCGAACAGCTGAGAGGACT
(2) CEBPα–(F) AAGGGTGTATGTAGTAGTGG; (R) AAAAAGAAGAGAAGGAAGCG
(3) LPL–(F) CATTGTAGTAGACTGGTTGTATCGGGC; (R) ATCTACAAAATCAGCGTCATCA
(4) ACT–(F) CATCGTGGGCCGCCCTAGGCACCA; (R) CGGTTGGCCTTAGGGTTCAGGGGG

### 4.7. Rheology and Viscoelastic Measurements

Cells were grown in GM or supplemented with MGO, GAD as described above. The cells were collected from the culture by scraping, centrifuged to a pellet at 2000 RPM, and the pellet air-dried. The pellet was measured for viscoelasticity to determine rheological properties using Discovery HR-3 hybrid Rheometer (TA Instruments, New Castle, DE, USA) on the 8-mm diameter parallel Peltier plate at 25 °C or 37 °C, according to the ISO 6721-1 method, as previously described [1,7]. The measured storage and loss modulus of the cells were monitored for G’, G” and Tan δ as a function of angular frequency either between 0.1–100 rad/s and a strain between 0.8–1 percent. An overtime test used a constant angular frequency of 1 rad/s at 37 °C to observe the transformation from liquid to solid-like behavior.

### 4.8. Mass Spectrometry

Mass spectrometry data were analyzed based on our database that was previously described [9].

### 4.9. Statistical Analysis

The statistical analysis of the experimental data was conducted using Microsoft Excel and GraphPad Prism 8. The results were presented as means ± standard deviation (SD). Statistical differences compared the mean values tested using two-tailed, unpaired t-tests (in the case of two groups). One way-ANOVA was used for three or more groups, followed by Sidak’s multiple comparison test. Values that were not normally distributed, were tested using the Kruskal–Wallis test (for three or more groups), followed by Dunn’s post-test for multiple comparisons. In the case of randomly missing values, the data were analyzed using the Mixed-effect analysis of variance, followed by Sidak’s multiple comparison test. Two-way ANOVA compared the mean difference between groups over different time points, followed by Sidak’s multiple comparison tests for statistical significance. A *p*-value < 0.05 was considered statistically significant. Statistical analysis and data plotting were performed using GraphPad Prism 8 software (La Jolla, CA, USA).

## Data Availability

Data are available from the authors upon reasonable request.

## Supplementary Materials

The supporting information can be downloaded at: https://www.mdpi.com/article/10.3390/ijms24032261/s1.
